# Data Mining and Network Pharmacology Characterize Medication Patterns of Chinese Herbal Medicine for Bovine Viral Diarrhea

**DOI:** 10.3390/vetsci13060575

**Published:** 2026-06-11

**Authors:** Miao An, Junhao Xiang, Huan Liu, Muhammed Farhan Rahim, Jiakui Li, Yiming Liu

**Affiliations:** 1 College of Veterinary Medicine, Huazhong Agricultural University, Wuhan 430070, China; 2 National Feed Drug Reference Laboratories, Feed Research Institute, Chinese Academy of Agricultural Sciences, Beijing 100089, China

**Keywords:** bovine viral diarrhea, traditional Chinese medicine, medication patterns, data mining, network pharmacology

## Abstract

The purpose of this study was to use data mining and network pharmacology to determine drug patterns for bovine viral diarrhea (BVD). The frequency, properties, tastes, meridian tropism, and functions of prescription data were gathered and examined from four literature databases (2004–2024). Cluster analysis was used to find prescription patterns, and the Apriori algorithm (SPSS Modeler 18) was used to find associations. A total of 391 literature-derived prescription records were included in the analysis, involving 189 distinct herbal medicines and a cumulative herb-use frequency of 2031 occurrences. These herbs primarily enter the liver meridian and were categorized as cold, warm, or neutral. The predominant tastes were bitter, pungent, and sweet. Five frequently recorded herbs and 14 co-occurrence patterns among herbs were extracted. According to traditional Chinese medicine (TCM), spleen–stomach deficiencies and damp-heat pathogens are linked to BVD. These medication patterns were mainly associated with heat-clearing, detoxification, spleen-strengthening, and Qi-regulating strategies in TCVM theory. Targets were screened, PPI networks were constructed, and enrichment studies for core herbs (Baitouweng, Huangbo, Huangqin, Qinpi, and Zhizi) were performed using network pharmacology. The binding affinities between disease targets and active components were further assessed using molecular docking. The findings provide a descriptive summary of medication patterns and generate preliminary hypotheses regarding potential compound–target–pathway associations involved in the symptomatic and supportive use of TCM for BVD.

## Simple Summary

Bovine viral diarrhea (BVD) is a problematic disease for cattle, and traditional Chinese herbal medicine is commonly used as a supportive approach for BVD-related clinical signs, but clear herbal medication patterns for this condition lack systematic organization. This study analyzed 391 literature-derived prescription records published between 2004 and 2024, comprising 189 herbs with a cumulative herb-use frequency of 2031 occurrences, using data mining and network pharmacology. The analysis characterized herb properties, tastes, meridian tropism, frequently recorded herbs, and co-occurrence patterns. Network pharmacology and molecular docking were further used to generate preliminary hypotheses on potential compound–target–pathway associations. These findings provide a descriptive overview of published herbal medication patterns for BVD and offer a basis for future experimental validation.

## 1. Introduction

Bovine viral diarrhea (BVD) is a disease caused by bovine viral diarrhea virus (BVDV), a member of the genus *Pestivirus* within the family *Flaviviridae*, and the major circulating genotypes are BVDV-1 and BVDV-2, although an atypical HoBi-like pestivirus has also been reported [1,2,3]. According to WOAH, BVDV infection has a broad clinical spectrum, including enteric, respiratory, reproductive, and fetal disease, as well as immunosuppression [4]. Therefore, gastrointestinal signs represent only one component of the disease spectrum rather than its sole or defining presentation, and BVDV-associated diarrhea should be distinguished from diarrhea caused by other enteric pathogens [5]. Respiratory signs in BVDV infection are often associated with immunosuppression and secondary infections. Therefore, diarrhea-related manifestations should be interpreted together with reproductive disorders, mucosal lesions, fetal infection, immunosuppression, and the possible presence of persistently infected (PI) animals. The central epidemiological feature of BVD is the generation of PI calves [6]. The prevalence of BVDV in Guangdong Province, China, was 2.03%, whereas markedly higher prevalence rates have been reported in other regions of the country [7]. For example, the seropositive rates of BVDV neutralizing antibodies in cattle from Hebei, Shandong, and Henan were 40.8%, 83.3%, and 53.8%, respectively [8]. Similar regional differences have also been observed in yak populations, with a BVDV prevalence of 67.5% reported in Xinjiang [9]. BVDV infection imposes a considerable economic burden on cattle production, with estimated annual losses of €42.14–67.19 per animal [10]. Over the past two decades, several fatal outbreaks of BVDV-associated mucosal disease have been reported in Argentina, and BVD-related abortions alone were estimated to cause economic losses of approximately $1,496,880,000 Argentine pesos, or about $100,000,000 [11]. Vaccination plays a critical role in strengthening herd immunity and decreasing the morbidity and mortality associated with BVDV infection in cattle. Evidence from 17 studies indicates that both modified-live vaccines (12 studies) and two-dose killed vaccines (5 studies) provide substantial clinical protection against experimental BVDV infection, reducing mortality by 80–100% and morbidity by 72–90% in calves [12]. However, the pathogenicity and genetic characteristics of circulating strains vary across regions, and such variability may weaken the cross-protective immunity induced by existing vaccines [13,14]. In addition, the PI animals, the difficulty in inducing effective immunity in already infected or immunotolerant animals, interference from maternal antibodies, and improper vaccination practices can also markedly compromise vaccine efficacy [15]. At present, there is no specific curative antiviral treatment for BVD, and disease management primarily relies on supportive therapy, prevention, vaccination, biosecurity, and the identification and removal of persistently infected animals [5]. Antibiotics may be used only when secondary bacterial infections are suspected or confirmed, but they do not target BVDV itself. In contrast, traditional Chinese medicine (TCM) is essentially symptomatic treatment and may alleviate certain clinical signs, but it cannot eliminate infection, remove PI animals, or prevent ongoing viral transmission and future mucosal disease outbreaks.

Traditional Chinese Veterinary Medicine (TCVM) may provide supportive value in the symptomatic management of BVD [16]. In clinical practice, Chinese herbal medicines (CHMs) are often used to alleviate gastrointestinal symptoms, reduce inflammatory responses, and promote recovery of the host’s functional status. Some such species are Isatis Tinctoria (Banlangen), Isatis Indigotica Fortune (Daqingye) and Reynoutria Japonica Houtt. (Huzhang) [17]. Second, other Chinese medicines can improve the host’s immunome, thus avoiding the entry of viruses into the cellular tissues; these herbs include Astragali Radix (Huangqi), Codonopsis Pilosula (Dangshen), and Poria (Fuling) [18,19]. CHMs typically contain multiple bioactive constituents, and they may act on several biological processes simultaneously, including inflammation, immune regulation, and tissue protection. This multicomponent and multitarget nature has led to increasing interest in their potential role as supportive interventions for complex infectious diseases. The growing body of published prescriptions and clinical records provides an opportunity to identify recurring medication patterns through data mining approaches [20,21,22]. In this study, “medication patterns” refer to literature-derived prescription characteristics, including herb frequency, properties, meridian tropism, co-occurrence relationships, and clustering features. These approaches can help reveal commonly recorded herbs and compatibility characteristics [23]. On this basis, network pharmacology offers a preliminary systems-level framework to explore the potential interactions among herbal compounds, targets, and disease-related pathways [24]. By integrating these approaches, it becomes possible to find medication patterns and to generate mechanistic hypotheses regarding the symptomatic and supportive use of CHMs in BVD.

Previous studies have mainly reported individual prescriptions or clinical observations, whereas systematic summaries of prescription-use characteristics remain limited. Therefore, this study used data mining to analyze herb frequency, properties, co-occurrence relationships, and clustering features in BVD-related TCM records. Network pharmacology and molecular docking were further applied as exploratory tools to generate hypotheses regarding potential compound–target–pathway associations. The study was designed to characterize literature-derived medication patterns.

## 2. Materials and Methods

### 2.1. Data Acquisition

We conducted a comprehensive literature search across four major databases: China National Knowledge Infrastructure (CNKI), Wanfang Data, China Science and Technology Journal (CSTJ), and PubMed. CNKI, Wanfang, and CSTJ were selected as the primary databases because most studies on Chinese herbal medicine for bovine viral diarrhea were published in Chinese-language journals. PubMed was additionally included to expand the retrieval scope and identify potentially relevant international publications [25]. The search encompassed publications in both Chinese and English, spanning from 1 January 2004 to 31 December 2024. Details of the search strategies and specific keywords used are provided in Table 1. The terms “diarrhea”, “damp-heat diarrhea”, and “compound” were retained to improve the sensitivity of the search, and all retrieved records were then manually screened for relevance to BVD.

### 2.2. Selection Criteria

#### 2.2.1. Inclusion Criteria

Studies published in Chinese or English between 1 January 2004 and 31 December 2024 were included if they reported TCM prescriptions for BVD or BVDV infection in cattle, involved single-herb or multi-herb formulations, used TCM alone, and provided extractable prescription data. Broad terms such as “diarrhea” and “damp-heat diarrhea” were used only at the retrieval stage to improve search sensitivity, because TCVM reports related to BVD may be indexed under symptom- or syndrome-based terms rather than under “BVD” alone. These terms were not used as independent inclusion criteria. During screening, records were retained only when the original publication explicitly described BVD or BVDV infection as the disease context. Studies reporting only nonspecific bovine diarrhea, damp-heat diarrhea, or diarrhea-like syndromes without a clear BVD/BVDV diagnosis were excluded. Because the included sources were mainly descriptive prescription reports rather than controlled intervention studies, conventional risk-of-bias tools for therapeutic efficacy were not directly applicable. This study emphasized predefined eligibility screening, standardized prescription extraction, and data normalization to improve the consistency of medication-pattern analysis.

#### 2.2.2. Exclusion Criteria

This analysis excluded studies involving the concurrent use of TCM and Western medicine, duplicate publications, mixed infections, and treatments for conditions similar to viral or bacterial diarrhea (including dysentery). When multiple studies reported the same therapeutic formula, only one instance was retained for analysis. Only studies in which the cases were explicitly identified as BVD or BVDV infection in the original reports were included. Because BVDV-induced immunosuppression may increase susceptibility to other enteric pathogens, studies in which diarrhea was mainly attributed to bacterial, parasitic, mixed, or nonspecific enteric infections rather than to BVD/BVDV infection were excluded.

### 2.3. Data Processing

#### 2.3.1. Data Pre-Processing

Based on the inclusion and exclusion criteria, the pertinent literature on the treatment of BVD using CMs was strategically collected. These articles were manually read with their prescriptions extracted and then put into the database in accordance with the standard practices. The prescriptions that failed to meet the outlined requirements were not included in the analysis. Three authors independently screened the retrieved records and extracted prescription information using a standardized form. Disagreements were resolved through discussion or consultation with a fourth author.

#### 2.3.2. Data Normalization and Entry

To overcome the inconsistency in TCM nomenclature, the herbal substances were standardized primarily based on the Veterinary Pharmacopeia of China, which served as the main reference for nomenclature normalization, while the Pharmacopeia of the People’s Republic of China (2020 Edition) was consulted as a supplementary reference when necessary. The functions and meridian affiliations of the herbs were typologized and the properties were organized. Because many of the included studies did not provide complete or standardized dosage-related information, dosage data could not be uniformly processed across studies and were therefore not included in the quantitative herbal prescription analysis.

#### 2.3.3. Statistical Analyses

**Figure 1:**
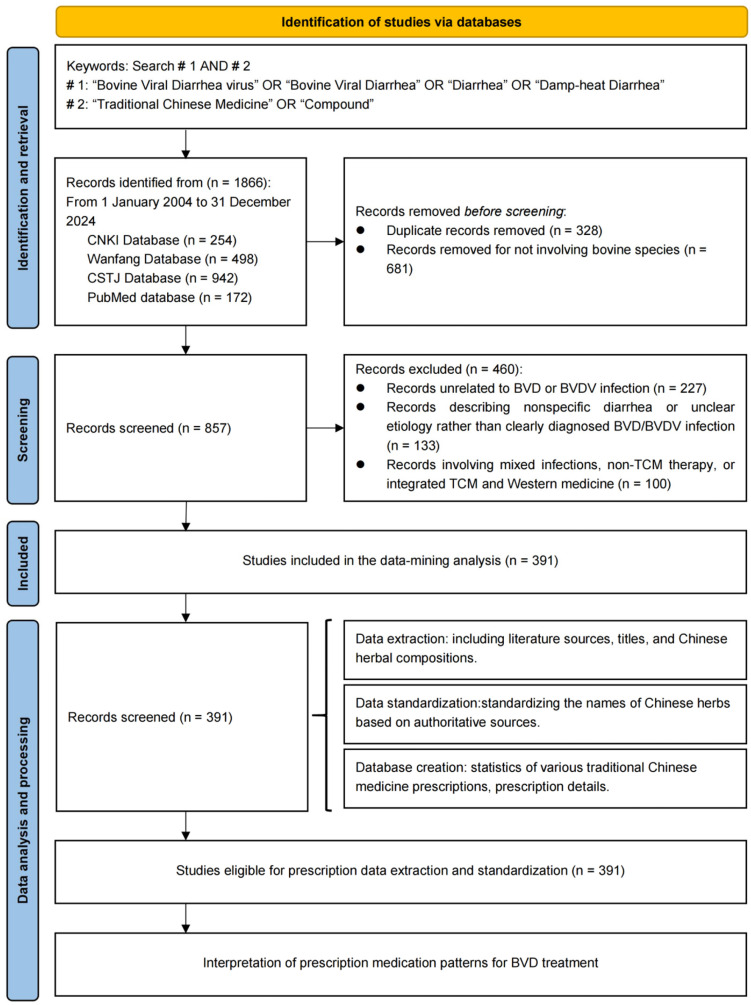
Screening process (PRISMA flow diagram).Note: “#” denotes the numbered search set used in the database search strategy. #1 indicates the BVD-related keyword set, and #2 indicates the traditional Chinese medicine-related keyword set. The final search strategy was generated by combining #1 and #2 with the Boolean operator AND.

A database was created using Endnote 20 and Microsoft Excel 2024 to catalog the literature, herbal prescriptions, and their compositions. The herbs were analyzed for their frequency of use, characteristics, meridian tropism, and functions. Initial statistical analyses were performed using pivot tables, and the data were visualized with bar charts and radar diagrams in Origin 2024. For the analysis of herb co-occurrence patterns, IBM SPSS Modeler version 18.0 was used to perform association rule mining with the Apriori algorithm. In association rule mining, support, confidence, and lift were used as the main parameters to evaluate the frequency and strength of herb co-occurrence [23]. Several threshold combinations with different support and confidence levels were compared during exploratory analysis. Support ≥0.10 and confidence ≥0.75 were selected to balance rule interpretability and information retention, while lift > 1.0 was used to retain only positively associated rules. Rules that satisfied all three criteria were retained for further interpretation. Cluster analysis was performed using IBM SPSS Statistics version 27.0 (squared Euclidean distance and between-groups linkage), and the results were visualized in Origin 2024. The literature screening process is shown in Figure 1.

### 2.4. Network Pharmacology in Conjunction with Molecular Docking

#### 2.4.1. Acquisition of Active Compounds and Disease-Related Targets

Potential active compounds and herb-related targets were obtained from the HERB database (http://herb.ac.cn/, accessed on 26 December 2025) and TCMSP database (https://www.tcmsp-e.com/load_intro.php?id=43, accessed on 26 December 2025). Only compounds with available target annotations were retained, and duplicates were removed based on compound names and PubChem IDs. BVD-related targets were collected from OMIM (https://omim.org/, accessed on 26 December 2025) and GeneCards (https://www.genecards.org/, accessed on 26 December 2025), and duplicate disease-related targets were removed before subsequent analysis. To improve species relevance, herb-related and disease-related protein targets were standardized using the UniProt database, mapped to bovine (*Bos taurus*) proteins where possible, and converted into standardized gene names [26]. The overlapping targets between herb-related targets and BVD-related targets were retained for subsequent analysis. Target selection based on HERB, TCMSP, OMIM, and GeneCards may preferentially capture well-annotated, high-connectivity, or non-specific targets, so the overlapping targets were interpreted as exploratory candidate nodes rather than validated BVD-specific candidate targets.

#### 2.4.2. PPI Network Construction

The overlapping herb-related and BVD-related targets were imported into the STRING (https://cn.string-db.org/, accessed on 28 December 2025) for protein–protein interaction (PPI) analysis, with the species set as *Bos taurus* when available. The resulting TSV file was subsequently visualized and analyzed using Cytoscape version 3.9.1. Within Cytoscape, the analysis of the structural characteristics was conducted using the parameters “degree”, “betweenness” and “closeness”. The network was subsequently visualized, and core targets were determined based on the values of these three centrality metrics.

#### 2.4.3. Enrichment Analysis

These targets were submitted to DAVID in preparation for enrichment analysis (GO and KEGG). To provide a hypothesis-generating interpretation of the prescription patterns, these analyses focused on biological processes and pathways potentially associated with the database-derived candidate targets. The species was specified as “Bovine”.

#### 2.4.4. Drug–Ingredient–Target–Disease Network and Molecular Docking

The collected herb–compound–target–disease information was systematically organized and imported into Cytoscape to construct the drug–ingredient–target–disease network. Three-dimensional SDF files of the herbal monomers were sourced from PubChem (https://pubchem.ncbi.nlm.nih.gov/, accessed on 5 February 2026), while the proteins’ PDB files were obtained from the AlphaFold (https://alphafold.com/, accessed on 5 February 2026). Pre-docking preparation, including energy minimization, deprotonation, and solvation, was carried out using PyMOL version 2.6. Because experimentally validated ligand-binding pockets were not available for most candidate targets, blind docking was performed by setting the grid box to cover the predicted protein structure. Subsequently, molecular docking simulations between the small-molecule compounds and target proteins were performed using AutoDock Vina version 1.5.6. Unless otherwise specified, default AutoDock Vina parameters were used. The predicted binding energy was used to evaluate compound–target affinity, and a lower binding energy was considered to indicate stronger predicted binding affinity.

## 3. Results

### 3.1. Data Mining on BVD

#### 3.1.1. Frequency Analysis

**Figure 2:**
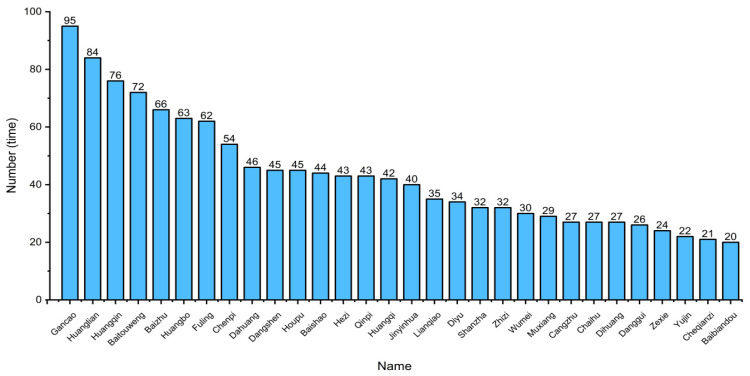
The frequency of high-frequency herb usage.

After screening, 391 of the 857 retrieved studies met the inclusion criteria. These included studies were treated as literature-derived prescription records for medication-pattern analysis, rather than as homogeneous clinical trials for evaluating therapeutic efficacy. The studies documented 189 distinct herbal medicines, resulting in a cumulative frequency of 2031 occurrences. Of the 189 identified herbal medicines, 30 (15.87%) were classified as high-frequency herbs, each appearing 20 times or more, and collectively accounting for 64.30% of the total cumulative frequency. Table 2 provides comprehensive data regarding the names, properties, tastes, meridian tropisms, functions, and usage frequencies of these high-frequency herbs. Subsequent citations of the herbs will be made using Chinese abbreviations. The five most frequently occurring herbal medicines, listed in descending order of frequency, are *Glycyrrhiza uralensis* (Gancao), *Coptis chinensis* (Huanglian), *Scutellaria baicalensis* (Huangqin), *Pulsatilla chinensis* (Baitouweng), and *Atractylodes macrocephala* (Baizhu) (Figure 2). These frequency results reflect repeated occurrence in the included prescriptions. Notably, several of these herbs overlap with those used in classical formulas for damp-heat diarrhea, such as Baitouweng Tang and Ge Gen Qin Lian Tang, which provides a traditional formula-based context.

#### 3.1.2. Analysis of Properties, Tastes, Meridian Affiliations, and Herbal Classification

**Figure 3:**
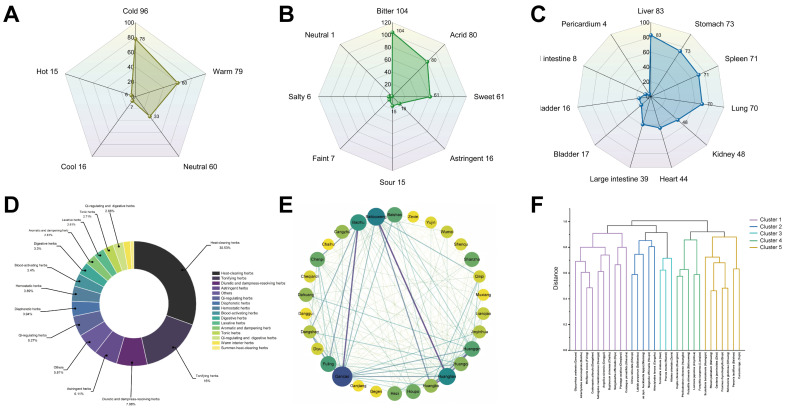
Data-mining analysis of BVD-related medication patterns. (**A**) Herb properties. (**B**) Herb tastes. (**C**) Meridian tropisms. (**D**) Functional categories of herbs. (**E**) Association network of high-frequency herbs. Nodes represent herbs, edges represent herb co-occurrence relationships, and edge width indicates association strength. (**F**) Cluster analysis of high-frequency herbs based on squared Euclidean distance and between-groups linkage. Different colors indicate different clusters.

This study undertook an analysis of the properties, meridian affinities and efficacy of 189 CHMs. Following the exclusion of data with indeterminate herbal characteristics, a total of 182 documented instances of herbal properties were subjected to evaluation. Of the instances evaluated, cold herbs were the most frequently identified, appearing 48 times (26.37%). This was followed by warm herbs (47 instances, 25.82%) and neutral herbs (33 instances, 18.13%). Herbs with a slightly cold quality were utilized on 28 occasions (15.38%), while slightly warm herbs were employed on 13 occasions (7.14%). In contrast, the use of cool herbs was documented on seven occasions (3.85%), hot herbs on five occasions (2.75%), very cold herbs on two occasions (1.10%), and very hot herbs on one occasion (0.55%), indicating a decreasing frequency of use (Figure 3A).

The frequencies and proportions of individual herbal categories are illustrated in Figure 3D. The most frequently utilized herbs were those with heat-clearing properties, which were employed on 620 occasions, representing 30.53% of the total usage frequency of 2031 instances. Subsequently, nourishing herbs were employed 344 times (21.37%), diuretic and dampness-resolving herbs were used 290 times (18.01%), astringent herbs were utilized 218 times (13.54%), and Qi-regulating herbs were used 107 times (5.27%).

#### 3.1.3. Association Rule Mining and Network Visualization

The Apriori algorithm (support ≥ 0.1, confidence ≥ 0.75, and lift > 1) was employed to examine the associations among 189 medicinal herbs, yielding 14 association rules. Additionally, an association rule network diagram was created using Cytoscape software to visualize these relationships. In the analysis of binary drug associations, the representative pairs identified were Huangqin and Zhizi, Gancao and Danggui, as well as Dahuang and Zhizi. The ternary drug association analysis revealed representative triplets, which included Baitouweng and Qinpi (Huangbo), Huangbo and Qinpi (Baitouweng), Gancao and Huangqi (Dangshen), Huanglian and Huangbo (Baitouweng), Huangqin and Zhizi (Dahuang), Huangbo and Baishao (Huangqin), Huanglian and Qinpi (Huangbo), Dahuang and Zhizi (Huangqin), Zhizi and Dahuang (Huangqin), and Huanglian and Qinpi (Baitouweng). The identified representative pairs were Huangqin and Zhizi (Dahuang), Huangbo and Baishao (Huangqin), Huanglian and Qinpi (Huangbo), Dahuang and Zhizi (Huangqin), Zhizi and Dahuang (Huangqin), and Huanglian and Qinpi (Baitouweng). Also, one representative combination was found through the quaternary drug association analysis, which is Huanglian and Qinpi (Huangbo, Baitouweng). The strongest associations were noted between Baitouweng, Qinpi and Huangbo, as shown in Table 3. In the network diagram, edge width is proportional to the association strength between herbs. The dataset was encoded using binary values (“0” and “1”). To ensure the reliability of the association rule analysis, only herbs that appeared 20 times or more were included. Based on this filtered data, the association networks were constructed and are illustrated in (Figure 3E). The results suggested that *Pulsatilla chinensis* (Baitouweng), *Phellodendron chinense* (Huangbo), *Scutellaria baicalensis* (Huangqin), *Gardenia jasminoides* (Zhizi), and *Fraxinus* spp. (Qinpi) were frequently co-recorded herbs in BVD-related TCM prescriptions.

#### 3.1.4. Cluster Analysis

Hierarchical cluster analysis of the 30 high-frequency herbs identified five clusters (Figure 3F). The first cluster (purple) was composed of the following herbs: *Glycyrrhiza uralensis* (Gancao), *Atractylodes macrocephala* (Baizhu), *Wolfiporia cocos* (Fuling), *Codonopsis pilosula* (Dangshen), and *Astragalus membranaceus* (Huangqi). The remaining herbs were *Angelica sinensis* (Danggui), *Bupleurum chinense* (Chaihu), *Sanguisorba officinalis* (Diyu), *Plantago asiatica* (Cheqianzi), and *Crataegus pinnatifida* (Shanzha). The second cluster (blue) was composed of *Citrus reticulata* (Chenpi), *Lablab purpureus* (Baibiandou), *Saussurea costus* (syn. *Aucklandia lappa*) (Muxiang), *Magnolia officinalis* (Houpo), and *Atractylodes lancea* (Cangzhu). Cluster 3 (cyan) comprised *Terminalia chebula* (Hezi), *Prunus mume* (Wumei), and *Alisma orientale* (Zexie). Cluster 4 (green) comprised *Coptis chinensis* (Huanglian), *Phellodendron chinense* (Huangbo), *Pulsatilla chinensis* (Baitouweng), *Lonicera japonica* (Jinyinhua), and *Forsythia suspensa* (Lianqiao). Cluster 5 (yellow) comprised the following herbs: *Scutellaria baicalensis* (Huangqin), *Rheum palmatum* (Dahuang), *Gardenia jasminoides* (Zhizi), *Fraxinus rhynchophylla* (Qinpi), *Rehmannia glutinosa* (Dihuang), *Paeonia lactiflora* (Baishao), and *Curcuma* spp. (Yujin). These clusters summarize commonly co-recorded herb groups in the prescription dataset and should not be interpreted as validated therapeutic combinations.

#### 3.1.5. Co-Occurrence-Based Candidate Herb Combination

Based on the drug pairs identified through association rules and the five types of herbs derived from systematic cluster analysis, and in accordance with the compatibility principle of “sovereign, minister, assistant and messenger”, a putative herb combination was summarized based on co-occurrence patterns, comprising Baitouweng, Huangbo, Huangqin, Qinpi, and Zhizi.

### 3.2. Network Pharmacological Analysis of Herbs on BVD

#### 3.2.1. Active Ingredients of Herbs and the Target of Herbs and Disease

Screening was performed using the TCMSP database, yielding a total of 81 components, including 9 from Baitouweng, 25 from Huangbo, 32 from Huangqin, 3 from Qinpi, and 12 from Zhizi. Additionally, 822 ingredients were collected from the HERB database, consisting of 108 from Baitouweng, 173 from Huangbo, 36 from Huangqin, 94 from Qinpi, and 222 from Zhizi. After compiling all the components and removing duplicates, 751 unique active ingredients were identified. Following the elimination of redundant entries, 444 targets were obtained. After removing duplicates from the disease targets sourced from relevant databases, a total of 3188 disease targets were identified.

#### 3.2.2. Acquisition of Intersection Targets and Construction of PPI Network

**Figure 4:**
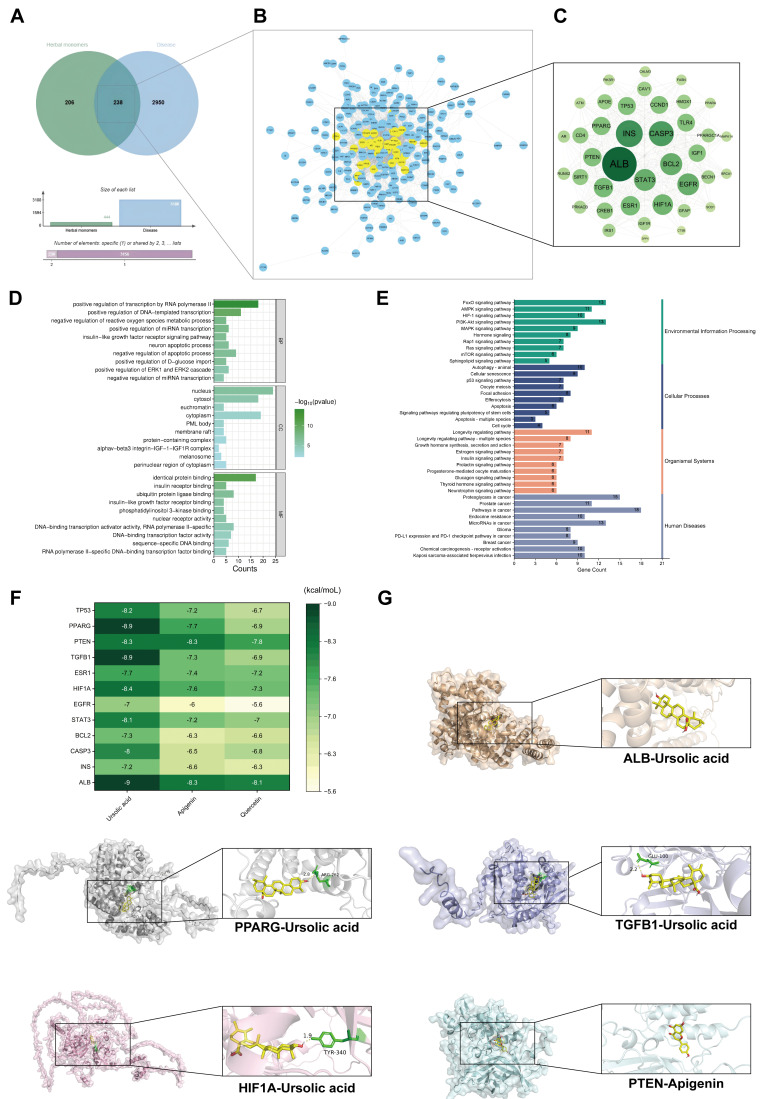
Network pharmacology and preliminary molecular docking analysis. (**A**) Venn diagram of active ingredient targets versus disease targets in Chinese medicine. (**B**) A total of 238 bovine-based protein networks. Nodes represent candidate targets, and edges represent predicted protein–protein interactions. (**C**) Topological screening of 39 core targets. Core targets were identified by topological analysis of the PPI network based on three centrality parameters: degree, betweenness centrality, and closeness centrality. Targets with degree centrality >21.19, betweenness centrality >299.76, and closeness centrality >0.0019 were retained as core targets. (**D**) GO functional enrichment analysis. (**E**) KEGG pathway enrichment analysis. (**F**,**G**) Preliminary molecular docking of selected compounds with candidate targets (Quercetin, Apigenin and Ursolic acid).

As illustrated in Figure 4A, the intersection between the drug-related targets and BVD-associated targets yielded a total of 238 common targets potentially involved in the symptomatic and supportive treatment of BVD by Baitouweng, Huangbo, Huangqin, Qinpi, and Zhizi. Following its construction in STRING and visualization in Cytoscape (Figure 4B), the overlapping target PPI network (231 nodes, 2447 edges) underwent topological screening. Using CentiScaPe 2.2, core targets were discerned through an assessment of key centrality metrics: betweenness, closeness, and degree. The final set of 39 potential core targets was chosen. Among these, the top-ranked hub targets by degree centrality included ALB, INS, CASP3, BCL2, STAT3, EGFR, HIF1A, ESR1, TGFB1, PPARG, PTEN, and TP53 (Figure 4C). These core targets represent highly interconnected candidate nodes in the constructed network, but many of them are broadly involved in general biological processes and have not been further experimentally validated.

#### 3.2.3. Functional and Pathway Module Characterization

The hypothetical candidate targets of Baitouweng, Huangbo, Huangqin, Qinpi, and Zhizi were subsequently profiled in DAVID for enrichment analysis. GO term enrichment analysis (Figure 4D) showed that the biological processes encompassed transcriptional regulation, cellular components were mainly the nucleus and cytosol, and molecular functions involved protein binding and insulin receptor binding. Pathway analysis revealed significant enrichment of the FoxO, PI3K-Akt, and AMPK signaling pathways (Figure 4E). These enrichment results provide preliminary clues to potential host-response pathways associated with the selected herbs, rather than confirming mechanisms of action in BVD.

#### 3.2.4. Preliminary Molecular Docking of Candidate Compounds and Targets

Molecular docking of the candidate compounds (Quercetin, PubChem ID: 5280343, Apigenin, PubChem ID: 5280443 and Ursolic acid, PubChem ID: 64945) with selected target proteins suggested potential binding affinities, with binding energies as low as −9.0 kcal/mol (Table 4, Figure 4F). Among these compounds, ursolic acid generally showed lower binding energies, suggesting stronger predicted affinity in the docking analysis. The most stable complex, visualized in Figure 4G, showed a predicted binding mode. However, molecular docking only predicts potential compound–target interactions and does not confirm biological activity, antiviral efficacy, or therapeutic effects in cattle.

#### 3.2.5. Multiscale Network Integration of Drug, Ingredient, Target, and Disease

**Figure 5:**
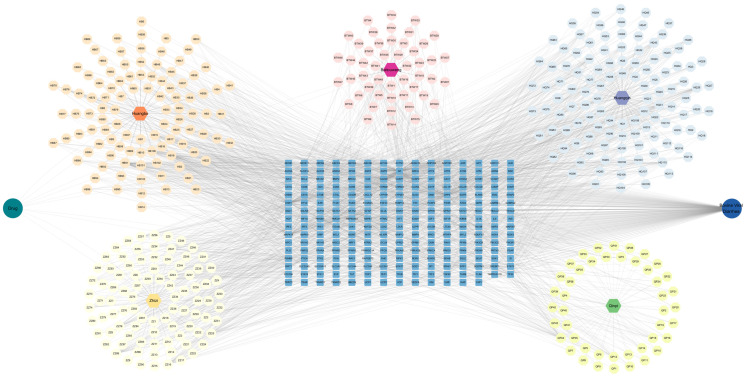
Drug–ingredient–target–disease network.

The herb–compound–target–disease network was constructed in Cytoscape to visualize predicted associations among herbs, compounds, candidate targets, and BVD-related terms (Figure 5).

## 4. Discussion

The diarrhea-related clinical manifestations of bovine viral diarrhea (BVD) are often interpreted within the framework of damp-heat diarrhea in Traditional Chinese Veterinary Medicine (TCVM) [27]. Within this theoretical framework, Chinese herbal medicine is often used as a supportive approach to alleviate diarrhea-related clinical manifestations and improve the general condition of affected animals. The main value of this study does not lie in re-emphasizing symptomatic treatment, but rather in systematically identifying medication patterns from prescription data and providing a hypothesis-generating interpretation of their possible literature-based relevance through network pharmacology. A notable strength of this study is that it moved beyond a simple description of individual herbs and instead analyzed the prescription corpus at multiple levels, including herb frequency, properties, associations, and clustering. This design allowed us to describe commonly recorded herbs, herb combinations, and the literature-based compatibility rationale that may underlie these co-occurrence patterns.

The five most frequently recorded herbs in BVD-related prescriptions were Gancao, Huanglian, Huangqin, Baitouweng, and Baizhu. These herbs were broadly consistent with the traditional principles of clearing heat, drying dampness, detoxification, and supporting spleen function in damp-heat diarrhea [28]. Interestingly, Gancao ranked first in frequency among all herbs. However, this should not indicate that it served as the principal therapeutic herb in most prescriptions. More likely, Gancao is widely recognized as a harmonizing and adjuvant herb in formula compatibility, helping to coordinate the actions of other herbs, moderate their properties, and support the overall effect of the prescription [29]. Therefore, the frequency results mainly indicate an overall prescription tendency characterized by heat-clearing, detoxifying, dampness-drying, and spleen-supporting strategies.

The drug combinations we identified showed substantial overlap with the herbal compositions of classical formulas for damp-heat diarrhea, particularly Baitouweng Tang (Pulsatilla Decoction) and Ge Gen Qin Lian Tang (Pueraria, Scutellaria, Coptis, and Licorice Decoction). The frequent inclusion of Qinpi and Zhizi may reflect possible modifications in modern veterinary applications. The commonly utilized herbs in this study were predominantly cold or warm in nature, mainly characterized by bitter, pungent, and sweet flavors, and frequently associated with the liver meridian, with bitter herbs traditionally interpreted as contributing to heat clearance, dampness drying, and detoxification [30]. These herb properties were also broadly consistent with their meridian tropism. The liver and spleen were central to the pathophysiology of damp-heat diarrhea [31,32]. Liver Qi stagnation impairs the spleen’s transporting and transforming functions, leading to the accumulation of internal damp-heat and triggering symptoms such as diarrhea and anal burning [33]. Conversely, spleen Qi deficiency hinders digestive function, exacerbating damp-heat conditions [34]. The herb combinations identified by association rule analysis showed correspondence with traditional medication patterns [35]. These association rules indicate co-occurrence patterns among herbs in the included prescriptions and may provide clues to the compatibility characteristics commonly described in BVD-related TCVM reports.

Cluster analysis further grouped the frequently used herbs into five categories. Cluster 1 included Gancao, Baizhu, Fuling, Dangshen, Huangqi, Danggui, Chaihu, Diyu, Cheqianzi, and Shanzha, which were mainly associated with tonifying Qi, strengthening the spleen, and supporting general physical condition. This pattern is partly consistent with formulas such as Buzhong Yiqi Decoction, which is commonly used for fatigue, poor appetite, Qi deficiency, and spleen weakness [36]. Cluster 2 included Chenpi, Baibiandou, Muxiang, Houpo, and Cangzhu, which resemble the core composition of Pingwei Powder and are mainly used to fortify the spleen, dry dampness, and regulate Qi stagnation in spleen–stomach damp obstruction [37]. Cluster 3 consisted of Hezi, Wumei, and Zexie, among which Wumei and Hezi are traditionally used for their astringent antidiarrheal effects [38]. Cluster 4 contained Huanglian, Huangbo, Baitouweng, Jinyinhua, and Lianqiao, a group characterized mainly by heat-clearing, dampness-drying, and detoxifying properties, and partly overlapping with the composition of Baitouweng injection [39]. Cluster 5 included Huangqin, Dahuang, Zhizi, Qinpi, Dihuang, Baishao, and Yujin, which were mainly associated with clearing heat, detoxification, purgation, blood cooling, and the relief of abdominal pain [40]. These clusters suggest that the prescriptions recorded in the literature may reflect multiple compatibility dimensions, including heat-clearing and detoxification, dampness resolution, bowel regulation, and support of spleen-Qi function.

To further explore the possible relevance of these medication patterns, BVD-related targets were integrated with targets associated with the five frequently recorded herbs, yielding 238 overlapping targets. Degree-based network analysis further identified several highly connected candidate nodes, including ALB, INS, and CASP3. However, these hub targets are broadly involved in multiple biological processes, and their identification mainly reflects network connectivity rather than confirmed disease-specific roles in BVD. The biological relevance of these targets should be interpreted cautiously and requires further experimental validation. Previous BVDV-related studies have reported associations with albumin stability, glucose metabolism or pancreatic function, and caspase-mediated lymphocyte apoptosis [41,42,43]. These observations provide indirect background for interpreting candidate targets identified by network analysis. Nevertheless, these targets should be regarded as literature- and database-derived hypotheses rather than confirmed BVD-specific mediators. GO analysis delineated notable enrichment in core biological processes, predominantly encompassing the positive modulation of RNA polymerase II and DNA-templated transcription. Cellular components were chiefly concentrated in the nucleus and cytosol, while molecular functions were predominantly enriched in identical protein binding and insulin receptor binding. KEGG pathway analysis showed enrichment of several broad signaling pathways, including cancer-associated pathways, FoxO, PI3K-Akt, and AMPK signaling pathways. The enrichment results should be interpreted as preliminary clues to potential host-response processes associated with the selected herbs, rather than as direct evidence of therapeutic mechanisms or antiviral activity against BVD. The PI3K-Akt pathway was among the enriched pathways, but this result should be interpreted only as a database-derived and hypothesis-generating clue. Previous studies in BVDV-related or non-BVD models have reported that quercetin, apigenin, and ursolic acid may affect oxidative stress, inflammatory signaling, apoptosis, autophagy, or PI3K/Akt-related pathways [44,45,46,47]. However, these findings provide only indirect background support and do not demonstrate that these compounds regulate PI3K-Akt signaling or exert antiviral effects in BVD-infected cattle. Therefore, this pathway is discussed as a candidate host-response pathway that requires further experimental validation.

Several limitations should be acknowledged. First, this study focused on prescription composition and compatibility patterns, but dosage information was not analyzed because standardized veterinary herbal medication databases covering dosage, prescription proportions, administration route, and treatment duration remain to be established. Second, because this study was based on published prescription records, diagnostic procedures in the original reports could not be retrospectively standardized. Third, a formal risk-of-bias assessment was not performed, as the included literature mainly consisted of descriptive prescription reports rather than controlled intervention studies. Therefore, the frequency, association rule, and clustering results should be interpreted as descriptive prescription-use patterns rather than evidence of clinical efficacy or standardized treatment recommendations.

From the perspective of BVD control, the therapeutic significance of these prescriptions should be interpreted cautiously, because symptomatic or supportive herbal treatment cannot eradicate BVDV infection, eliminate persistently infected animals, or prevent the birth of PI calves. In addition, target selection may be influenced by database annotation bias, which can overrepresent well-studied or highly connected proteins. The compound–target–pathway relationships were inferred from databases, network analysis, and molecular docking without in vitro, in vivo, or clinical validation. Thus, the proposed mechanisms should be regarded as preliminary hypotheses rather than confirmed biological conclusions. Future studies should validate candidate herbal compounds and prescriptions using BVDV-infected bovine cell models and confirmed BVDV-infected cattle to clarify their practical value as symptomatic and supportive interventions.

## 5. Conclusions

To conclude, this study used data mining and network pharmacology to characterize literature-derived medication patterns of Chinese herbal medicine for BVD. Cold-natured herbs, particularly those associated with the liver meridian, were frequently recorded in the included prescriptions. Co-occurrence and clustering analyses further suggested several commonly recorded compatibility characteristics in BVD-related TCM records. Network pharmacology and molecular docking provided hypotheses regarding potential compound–target–pathway associations, which may help inform future mechanistic studies. Overall, this study offers a descriptive framework for understanding published herbal prescription patterns related to BVD, but experimental and clinical validation is still required.

## Figures and Tables

**Table 1:** Overview of the literature search strategy across databases.

Keywords	Elements
#1	“Bovine Viral Diarrhea virus” OR “Bovine Viral Diarrhea” OR “Diarrhea” OR “Damp-heat Diarrhea”
#2	“Traditional Chinese Medicine” OR “Compound”
#3	Search #1 AND #2

Note: The symbol “#” denotes the numbered search set used in the database search strategy. #1 and #2 indicate separate keyword sets, while #3 indicates the combined search query generated by applying the Boolean operator AND between #1 and #2.

**Table 2:** Commonly used high-frequency herbs in the treatment of BVD.

No.	Herb	Properties	Tastes	Meridian Tropisms	Functionalities	Frequency ^1^	Proportion/% ^2^
1	*Glycyrrhiza uralensis* (Gancao)	Neutral	Sweet	Lung/Spleen/Stomach/Heart	Tonifying herbs	95	7.27%
2	*Coptis chinensis* (Huanglian)	Cold	Bitter	Large intestine/Small intestine/Gallbladder/Liver/Spleen/Stomach/Heart	Heat-clearing herbs	84	6.43%
3	*Scutellaria baicalensis* (Huangqin)	Cold	Bitter	Large intestine/Small intestine/Gallbladder/Lung/Spleen/Small intestine	Heat-clearing herbs	76	5.82%
4	*Pulsatilla chinensis* (Baitouweng)	Cold	Bitter	Stomach/Large intestine/Small intestine	Heat-clearing herbs	72	5.51%
5	*Atractylodes macrocephala* (Baizhu)	Warm	Sweet/Bitter	Spleen/Stomach	Tonifying herbs	66	5.05%
6	*Phellodendron chinense* (Huangbo)	Cold	Bitter	Bladder/Kidney	Heat-clearing herbs	63	4.82%
7	*Wolfiporia cocos* (Fuling)	Neutral	Sweet/Faint	Lung/Spleen/Kidney/Heart	Diuretic and dampness-resolving herbs	62	4.75%
8	*Citrus reticulata* (Chenpi)	Warm	Acrid/Bitter	Lung/Spleen	Qi-regulating herbs and digestive herbs	54	4.13%
9	*Rheum palmatum* (Dahuang)	Cold	Bitter	Large intestine/Small intestine/Liver/Spleen/Stomach/Pericardium	Laxative herbs	46	3.52%
10	*Codonopsis pilosula* (Dangshen)	Neutral	Sweet	Lung/Spleen	Tonifying herbs	45	3.45%
11	*Magnolia officinalis* (Houpo)	Warm	Acrid/Salty	Bladder	Qi-regulating herbs	45	3.45%
12	*Paeonia lactiflora* (Baishao)	Slightly Cold	Bitter/Sour	Liver/Spleen	Tonifying herbs	44	3.37%
13	*Terminalia chebula* (Hezi)	Neutral	Bitter/Sour/Astringent	Large intestine/Small intestine/Lung	Astringent herbs	43	3.29%
14	*Fraxinus rhynchophylla* (Qinpi)	Cold	Bitter/Astringent	Large intestine/Small intestine/Gallbladder/Liver	Heat-clearing herbs	43	3.29%
15	*Astragalus membranaceus* (Huangqi)	Warm	Sweet	Lung/Spleen	Nutritive herbs	42	3.22%
16	*Lonicera japonica* (Jinyinhua)	Cold	Sweet	Lung/Stomach/Heart	Heat-clearing herbs	40	3.06%
17	*Forsythia suspensa* (Lianqiao)	Slightly Cold	Bitter	Lung/Heart/Small intestine	Heat-clearing herbs	35	2.68%
18	*Sanguisorba officinalis* (Diyu)	Slightly Cold	Bitter/Sour/Astringent	Large intestine/Small intestine/Liver	Hemostatic herbs	34	2.60%
19	*Crataegus pinnatifida* (Shanzha)	Slightly Warm	Sweet/Sour	Liver/Spleen/Stomach	Digestive herbs	32	2.45%
20	*Gardenia jasminoides* (Zhizi)	Neutral	Sweet	Heart/Kidney/Large intestine/Small intestine	Heat-clearing herbs	32	2.45%
21	*Prunus mume* (Wumei)	Neutral	Sour/Astringent	Large intestine/Small intestine/Lung/Liver/Spleen	Astringent herbs	30	2.30%
22	*Saussurea costus* (syn. *Aucklandia lappa*) (Muxiang)	Warm	Acrid	Gallbladder/Liver/Pericardium	Qi-regulating herbs	29	2.22%
23	*Atractylodes lancea* (Cangzhu)	Warm	Acrid/Bitter	Liver/Spleen/Stomach	Aromatic and dampening herb	27	2.07%
24	*Bupleurum chinense* (Chaihu)	Slightly Cold	Acrid/Bitter	Gallbladder/Lung/Liver	Diaphoretics	27	2.07%
25	*Rehmannia glutinosa* (Dihuang)	Cold	Sweet/Bitter	Heart/Liver/Kidney	Heat-clearing herbs	27	2.07%
26	*Angelica sinensis* (Danggui)	Warm	Sweet/Acrid	Liver/Spleen/Heart	Tonifying herbs	26	1.99%
27	*Alisma orientale* (Zexie)	Cold	Sweet/Faint	Bladder/Kidney	Diuretic and dampness-resolving herbs	24	1.84%
28	*Curcuma* spp. (Yujin)	Cold	Acrid/Bitter	Lung/Liver/Heart	Blood-activating herbs	22	1.68%
29	*Plantago asiatica* (Cheqianzi)	Slightly Cold	Sweet	Lung/Liver/Kidney/Small intestine	Diuretic and dampness-resolving herbs	21	1.61%
30	*Lablab purpureus* (Baibiandou)	Slightly Warm	Sweet	Spleen/Stomach	Summer-heat-clearing herbs	20	1.53%

^1^ Frequency refers to the number of occurrences of each herb in the included prescriptions. ^2^ Proportion (%) was calculated as frequency/total frequency × 100%.

**Table 3:** Association rules of CHMs for BVD.

No.	Former Item	Latter Item	Support/%	Confidence/%	Lift
1	*Pulsatilla chinensis* (Baitouweng)	*Fraxinus rhynchophylla* (Qinpi) and *Phellodendron chinense* (Huangbo)	11.11	**92.31** ^1^	3.00
2	*Phellodendron chinense* (Huangbo)	*Fraxinus rhynchophylla* (Qinpi) and *Pulsatilla chinensis* (Baitouweng)	11.97	85.71	3.18
3	*Glycyrrhiza uralensis* (Gancao)	*Astragalus membranaceus* (Huangqi) and *Codonopsis pilosula* (Dangshen)	10.68	84.00	2.07
4	*Coptis chinensis* (Huanglian)	*Phellodendron chinense* (Huangbo) and *Pulsatilla chinensis* (Baitouweng)	**15.38** ^1^	80.56	2.24
5	*Scutellaria baicalensis* (Huangqin)	*Gardenia jasminoides* (Zhizi) and *Rheum palmatum* (Dahuang)	10.26	79.17	2.44
6	*Phellodendron chinense* (Huangbo)	*Paeonia lactiflora* (Baishao) and *Scutellaria baicalensis* (Huangqin)	10.26	79.17	2.94
7	*Coptis chinensis* (Huanglian)	*Fraxinus rhynchophylla* (Qinpi), *Phellodendron chinense* (Huangbo) and *Pulsatilla chinensis* (Baitouweng)	10.26	79.17	2.21
8	*Scutellaria baicalensis* (Huangqin)	*Gardenia jasminoides* (Zhizi)	13.68	78.13	2.41
9	*Glycyrrhiza uralensis* (Gancao)	*Angelica sinensis* (Danggui)	11.11	76.92	1.89
10	*Coptis chinensis* (Huanglian)	*Fraxinus rhynchophylla* (Qinpi) and *Phellodendron chinense* (Huangbo)	11.11	76.92	2.14
11	*Rheum palmatum* (Dahuang)	*Gardenia jasminoides* (Zhizi) and *Scutellaria baicalensis* (Huangqin)	10.68	76.00	3.95
12	*Gardenia jasminoides* (Zhizi)	*Rheum palmatum* (Dahuang) and *Scutellaria baicalensis* (Huangqin)	10.68	76.00	5.56
13	*Rheum palmatum* (Dahuang)	*Gardenia jasminoides* (Zhizi)	13.68	75.00	3.90
14	*Coptis chinensis* (Huanglian)	*Fraxinus rhynchophylla* (Qinpi) and *Pulsatilla chinensis* (Baitouweng)	11.97	75.00	2.09

^1^ Bolded numbers indicate the maximum value in the corresponding column.

**Table 4:** The relevant information and docking scores of compounds and proteins.

Target	Target ID	Compounds	Binding Energy (kcal/mol)
Albumin (ALB)	AF–P02769–F1	Ursolic acid	−9.0
		Apigenin	−8.3
		Quercetin	−8.1
Apoptosis regulator Bcl–2 (BCL2)	AF–O02718–F1	Ursolic acid	−7.3
		Quercetin	−6.6
		Apigenin	−6.3
Caspase 3 (CASP3)	AF–A0AAA9SVK2–F1	Ursolic acid	−8.0
		Quercetin	−6.8
		Apigenin	−6.5
Epidermal growth factor receptor (EGFR)	AF–Q6RXL6–F1	Ursolic acid	−7.0
		Apigenin	−6.0
		Quercetin	−5.6
Estrogen receptor 1 (ESR1)	AF–P49884–F1	Ursolic acid	−7.7
		Apigenin	−7.4
		Quercetin	−7.2
Hypoxia-inducible factor 1–alpha (HIF1A)	AF–Q9XTA5–F1	Ursolic acid	−8.4
		Apigenin	−7.6
		Quercetin	−7.3
Insulin (INS)	AF–P01317–F1	Ursolic acid	−7.2
		Apigenin	−6.6
		Quercetin	−6.3
Peroxisome proliferator–activated receptor gamma (PPARG)	AF–O18971–F1	Ursolic acid	−8.9
		Apigenin	−7.7
		Quercetin	−6.9
Phosphatase and tensin homolog (PTEN)	AF–A0A3Q1MXQ5–F1	Apigenin	−8.3
		Ursolic acid	−8.3
		Quercetin	−7.8
Signal transducer and activator of transcription 3 (STAT3)	AF–P61635–F1	Ursolic acid	−8.1
		Apigenin	−7.2
		Quercetin	−7.0
Transforming growth factor beta 1 (TGFB1)	AF–P18341–F1	Ursolic acid	−8.9
		Apigenin	−7.3
		Quercetin	−6.9
Tumor protein p53 (TP53)	AF–P67939–F1	Ursolic acid	−8.2
		Apigenin	−7.2
		Quercetin	−6.7

## Data Availability

The data presented in this study are available in Zenodo at https://doi.org/10.5281/zenodo.20527887, reference number 20527887. The literature records and extracted prescription data were derived from the following resources available in the public domain: China National Knowledge Infrastructure (CNKI, https://www.cnki.net/), Wanfang Data (https://www.wanfangdata.com.cn/), China Science and Technology Journal (CSTJ, https://www.cqvip.com/), and PubMed (https://pubmed.ncbi.nlm.nih.gov/).
